# Virtual clinical trial-based study for clinical evaluation of projection-reduced low-dose cone-beam CT for image guided radiotherapy

**DOI:** 10.3389/fonc.2024.1369603

**Published:** 2024-07-05

**Authors:** Meijiao Wang, Kaining Yao, Yixin Zhao, Jianhao Geng, Xianggao Zhu, Zhiyan Liu, Yongheng Li, Hao Wu, Yi Du

**Affiliations:** ^1^ Key Laboratory of Carcinogenesis and Translational Research (Ministry of Education/Beijing), Department of Radiation Oncology, Peking University Cancer Hospital & Institute, Beijing, China; ^2^ Department of Otolaryngology, Head and Neck Surgery, Peking University People’s Hospital, Beijing, China; ^3^ Institute of Medical Technology, Peking University Health Science Center, Beijing, China

**Keywords:** CBCT, low dose, image guided radiotherapy, virtual clinical trial, imaging dose

## Abstract

**Purpose:**

Repeated cone-beam CT (CBCT) scans for image-guided radiotherapy (IGRT) increase the health risk of radiation-induced malignancies. Patient-enrolled studies to optimize scan protocols are inadequate. We proposed a virtual clinical trial-based approach to evaluate projection-reduced low-dose CBCT for IGRT.

**Materials and methods:**

A total of 71 patients were virtually enrolled with 26 head, 23 thorax and 22 pelvis scans. Projection numbers of full-dose CBCT scans were reduced to 1/2, 1/4, and 1/8 of the original to simulate low-dose scans. Contrast-to-noise ratio (CNR) values in fat and muscle were measured in the full-dose and low-dose images. CBCT images were registered to planning CT to derive 6-degree-of-freedom couch shifts. Registration errors were statistically analyzed with the Wilcoxon paired signed-rank test.

**Results:**

As projection numbers were reduced, CNR values descended and the magnitude of registration errors increased. The mean CNR values of full-dose and half-dose CBCT were >3.0. For full-dose and low-dose CBCT (i.e. 1/2, 1/4 and 1/8 full-dose), the mean registration errors were< ± 0.4 mm in translational directions (LAT, LNG, VRT) and ±0.2 degree in rotational directions (Pitch, Roll, Yaw); the mean magnitude of registration errors were< 1 mm in translation and< 0.5 degree in rotation. The couch shift differences between full-dose and low-dose CBCT were not statistically significant (p>0.05) in all the directions.

**Conclusion:**

The results indicate that while the impact of dose-reduction on CBCT couch shifts is not significant, the impact on CNR values is significant. Further validation on optimizing CBCT imaging dose is required.

## 1 Introduction

On-board cone-beam computed tomography (CBCT) plays a considerable role in modern radiotherapy, as it is able to provide three-dimensional anatomy images with high-resolution. The use of CBCT facilitates patient positioning verification and target localization by anatomy alignment with reference to planning CT images, therefore substantially improving the accuracy, safety and effectiveness of image-guided radiotherapy (IGRT) (1–3). Despite the numerous advantages, CBCT comes at increased x-ray exposure compared with 2D radiographic imaging (4, 5). Since patients mostly receive recurrent CBCT scans over the entire treatment course, the health risk of imaging radiation induced by CBCT has raised serious concerns (6–11). For instance, it was estimated by Zhou et al. (8) that the average lifetime risk of cancer attributed to cumulative imaging doses per 100,000 individuals was 78 for brain cancer, 271 for lung cancer, and 510 for leukemia. A recent international survey on imaging practices in radiotherapy (12) shows that most centers used vendor-provided CBCT protocols in routine practice and less than 50% optimize the protocols to some extent. Growing efforts have been made to reduce imaging dose, and several strategies are recommended (4, 13, 14). A common and clinically available strategy of dose reduction is to reduce x-ray exposure via three techniques. The first technique is to use a short-arc scan over about 200 degrees instead of a 360-degree full-arc scan. The data acquired over short arcs are sufficient to reconstruct images of small objects, and therefore short-arc configuration has become the default for head-and-neck and pediatric protocols. The second is to decrease tube output (mAs) so as to reduce incident x-ray photons per projection, and the third is to increase angular separation to reduce exposure frequency. Several studies demonstrate that reduced exposure leads to CBCT image quality degradation. Wood et al. (15) evaluated mAs reduction on a pelvis phantom and made size-based recommendation of mAs settings. Yan et al. (16) investigated the relationship between the image quality and imaging dose and established a mAs-dependent empirical function. Lu et al. (17) evaluated the effect of reducing projection number on image quality and registration accuracy, concluding the projection-number reduction technique could achieve uncompromised registration accuracy under conscientious care. Similarly, Men et al. (18) evaluated the image quality, dose and registration errors acquired with different CBCT angular separations, indicating that CBCT images with fewer projections impair image quality and registration accuracy. Olch et al. (19) compared the image quality and registration accuracy between kV-pair imaging and low-dose CBCT acquired with reduced mAs and projections. The study demonstrates that CBCT dose can be reduced to a level comparable to kV-pair imaging with similar or enhanced patient positioning accuracy.

It is important to note that all the aforementioned studies are all phantom-based without any patient-involved validation. To our knowledge, there were only two relevant clinical study on low-dose CBCT for IGRT. The first study was by Alcorn et al. (20), which was an international study to evaluate the feasibility of a pre-defined low-dose CBCT protocol in pediatrics that received central neural system radiotherapy. The other study was by Bryce-Atkinson et al. (21) using CBCT scans from 7 pediatric patients to simulate low-dose scans by either adding noise or halving the number of projections. Due to the limited patient category, imaging site and cohort size, further validation is required. To this end, this study aims to comprehensively evaluate the image quality, image dose, and registration accuracy of CBCT images reconstructed using projections acquired with different combinations of angular range. The key innovation and highlight are: 1) we used a virtual clinical trial-based approach retrospectively enrolling a total of 71 real patients, and 2) we evaluated the clinical viability of low-dose CBCT for head, thorax and pelvis IGRT.

## 2 Materials and methods

### 2.1 Patient enrollment and data acquisition

A total of 71 patients treated at our institution (Peking University Cancer Hospital, China) were retrospectively enrolled under the approval of the institutional review board. The patients were all adults (age ranging from 27 to 68) without any metal implant and were treated on either an Edge linac or VitalBeam linac (Varian Medical Systems, Palo Alto, USA) from March 2022 to July 2022. The patients were categorized into three groups according to imaging sites, i.e., 26 head scan patients (10 with brain metastasis, 9 with nasopharyngeal carcinoma and 7 with glioma), 23 thorax scan patients (all with lung cancer) and 22 pelvis scan patients (9 with cervical cancer, 9 with rectal cancer and 4 with liver cancer). For the head scan group, patients were all immobilized with the open-face Double-Shell Positioning System (MacroMedics, Belgium) (22). For the thorax scan group, 15 patients were immobilized with thermoplastic and the other 8 with vacuum bag cushions. For the pelvis scan group, 4 liver patients were immobilized with ZiFix™ abdominal compression belt (Qfix, USA).

The pertinent imaging protocols were listed in **Table 1** (23). For each patient, the raw scan data of the first CBCT scan was off-line retrieved from the linac imaging node. The CBCT imaging dose was defined in weighted CT dose index (CTDIw), and was recalibrated previous to this study with the aid of on-site Varian service team.

**Table 1:** Key parameters of default (full-dose) CBCT protocols.

Protocol	kVp	Scan Range (deg)	Projection Number	Exposure (mAs)
Head	100	200	500	150
Thorax	125	360	895	270
Pelvis	125	360	895	270

### 2.2 Generation of projection-reduced low-dose CBCT

For each clinical CBCT scan, three low-dose scans were derived by picking the first projection of every two, four and eight projections to simulate 1/2, 1/4 and 1/8 the clinical sampling rate. This data resampling approach was to simulate a virtual clinical trial, in which a patient was simultaneously scanned at four dose levels, i.e., default (full-dose), 1/2, 1/4, and 1/8 the full dose levels. Therefore, the scan series were referred to as *S_1_*, *S_1/2_*, *S_1/4_*, and *S_1/8_*, respectively.

All the scan data were preprocessed and reconstructed in MATLAB R2022b (Mathworks, USA) using TIGRE-VarianCBCT, a validated open-source toolkit for on-board CBCT imaging (24). The reconstruction algorithm was FDK (Feldkamp, Davis and Kress), which is identical to the algorithm used in the current state of clinical practice.

### 2.3 Impact evaluation

#### 2.3.1 Soft tissue contrast

The soft tissue contrast was a key aspect to reflect image quality. Herein, we adopted the muscle and fat contrast-to-noise (CNR) proposed by Bryce-Atkinson et al. (21) to define the soft-tissue contrast in Equation 1 as


(1) $$\text{CNR}=\frac{\left|{\text{M}}_{\text{muscle}}-{\text{M}}_{\text{fat}}\right|}{\sqrt{{\text{σ}}_{\text{muscle}}^{2}+{\text{σ}}_{\text{fat}}^{2}}}$$

where M and σ were the mean HU value and standard deviation over the selected region-of-interest (ROI). In this study, the soft tissue contrast performance was defined as acceptable if the CNR exceeded 3 as per the Rose criterion (25), in line with the study by Bryce-Atkinson et al. (21).

For the head scan patients, the ROI was defined at the nasopharynx level: lateral pterygoid was selected as the muscle region, and the subcutaneous fat was fat region, as shown in **Figure 2A**. For the thorax and pelvis scan patients, subcutaneous fat and muscle regions were selected as shown in **Figures 2B, C**.

#### 2.3.2 Relative registration errors

The CBCT images of different dose levels were rigidly registered to planning CT to acquire couch shifts. The registration was performed in 3D Slicer (version 5.2.1) (26), open source software application for medical image analysis. While 3D Slicer was not intended for clinical use, the key reason we used it was that the registration algorithm (mutual information based rigid registration) in 3D Slicer was identical with the clinical system. The CBCT-to-CT registration was rigid and in 6-degree-of-freedom (6DoF), i.e., three translational directions (VRT, LAT and LNG) and three rotational directions (Yaw, Pitch and Roll). The registration result of the full-dose level (S_1_) was used as gold standard benchmark, and registration accuracy between the low-dose CBCT scans and the respective benchmark were compared to derive relative registration errors in each direction. The magnitude of the relative registration errors were calculated in Equations 2, 3 as


(2) $$\text{Tx}=\sqrt{{\text{ε}}_{\text{VRT}}^{2}+{\text{ε}}_{\text{LAT}}^{2}+{\text{ε}}_{\text{LNG}}^{2}}$$

(3) $$\text{Rx}=\sqrt{{\text{ε}}_{\text{Yaw}}^{2}+{\text{ε}}_{\text{Pitch}}^{2}+{\text{ε}}_{\text{Roll}}^{2}}$$

where ε was the relative registration error, and Tx and Rx were the magnitudes of translational and rotational relative errors respectively.

#### 2.3.3 Statistical analysis

The mean, standard deviation, 25–75% percentile (Q1-Q3), minimum and maximum values over the CNR results and relative registration errors were calculated. In addition, the 6DoF couch shifts acquired by registration to planning-CT via different CBCT image series were compared. The couch shifts of S_1/2_, S_1/4_ and S_1/8_ were paired with the full-dose method in each direction and then tested with the Wilcoxon paired signed-rank test. In this study, data analysis was performed in OriginPro (version 2021a, OriginLab, USA), and p-value< 0.05 was considered statistically significant.

## 3 Results

**Figure 1:**
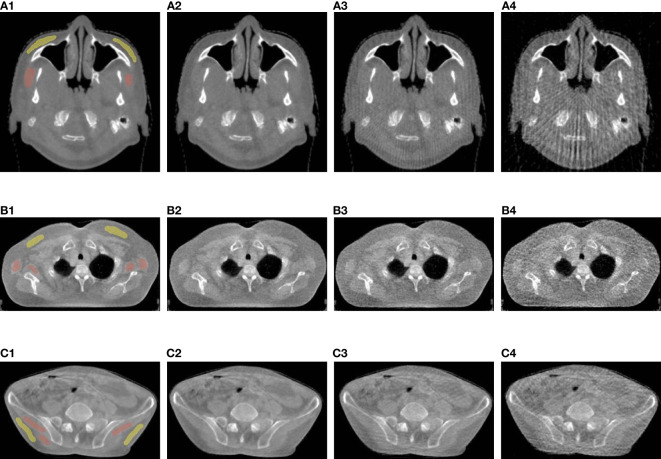
Visual image quality comparison of head (**A**-row), thorax (**B**-row), and pelvis (**C**-row) CBCT. The 1^st^ column shows full-dose images, and 2^nd^, 3^rd^ and 4^th^ columns show *S_1/2_*, *S_1/4_*, and *S_1/8_* images. The red and yellow annotations represent subcutaneous fat and muscle regions for CNR calculation.

**Figure 1** shows representative reconstructed images of real patients that received head (a-row), thorax (b-row) and pelvis (c-row) scans respectively with the default (*S_1_*) and projection-reduced protocols (*S_1/2_, S_1/4_, S_1/8_*). As we reduced the projection number, image quality deteriorated as the dose was lowered. Compared with the full-dose reference images (left-column in **Figure 1**), image noise and strike artifacts in dose-reduced protocols became obvious, which were induced by increased angular separations. While image details were blurred, the loss of soft-tissue contrast was relatively greater than that of bony structures.

**Figure 2:**
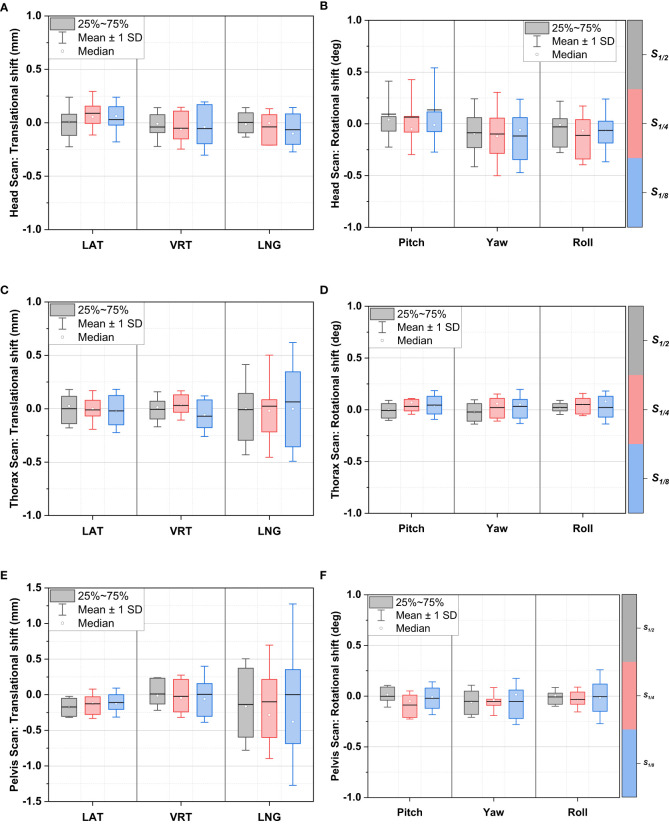
Relative registration error distributions between projection-reduced CBCT and full-dose CBCT in 6DoF: head scan translational shifts **(A)** and rotational shifts **(B)**; thorax scan translational shifts **(C)** and rotational shifts **(D)**; pelvis scan translational shifts **(E)** and rotational shifts **(F)**.

The 6DoF registration errors of projection-reduced head, thorax, and pelvis scans relative to the full-dose scans are summarized in **Figure 2**, showing the median, mean ± standard deviation (SD) and 25–75% interpercentile range (Q1-Q3). We can clearly see that, regardless of imaging site or scan protocol, the mean and median values of relative errors across six directions were quite close to zero, ranging within ±0.4 mm in translation and within ±0.2 degrees in rotation. We use Q1-Q3 and mean ± SD to indicate uncertainties in relative errors. For translational directions, the Q1-Q3 values across all protocols ranged within ±0.5 mm in translation except that in LNG for pelvis scan protocols (± 0.7 mm), and a similar trend was identified for mean ± SD. For rotational directions, the uncertainties in head protocols were relatively larger than in those in thorax and pelvis protocols. This may be attributed to the uncertainties induced by cervical/neck flexion. In addition, the couch shifts of *S_1/2_, S_1/4_* and *S_1/8_* were paired with those of *S_1_* in each direction to perform Wilcoxon paired signed-rank test. The p-values across all the tests were > 0.05, among which the smallest value was about 0.17, indicating the couch shift differences between projection-reduced protocols and the full-dose protocol were not statistically significant.

**Figure 3:**
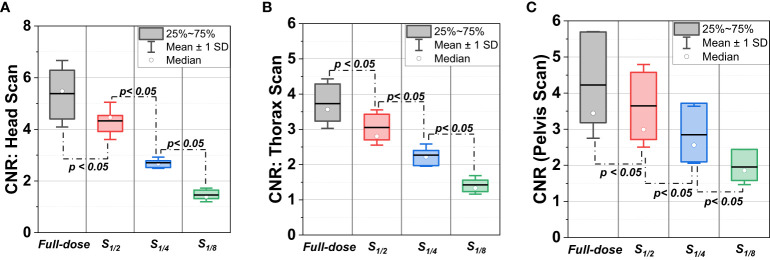
CNR distributions of full-dose CBCT scans and low-dose CBCT scans (*S_1/2_*, *S_1/4_*, and *S_1/8_*) in sites of **(A)** head, **(B)** thorax and **(C)** pelvis.

The CNR, Tx and Rx values over the testing patients were summarized in **Table 2** and **Figure 3**. As the projection numbers were reduced, the imaging dose decreased, leading to significant descending in CNR performance and ascending in Tx and Rx values. For CNR, while the *S_1/2_* protocol exhibited inferior performance to the full-dose protocol, the mean values across the three sites were >3.0, indicating the soft-tissue contrast performance was generally acceptable even when the projection number was halved from the default setting. For Tx, the mean values were mostly less than 1 mm except for *S_1/8_* protocol in pelvis scan. This is similar to the trend in **Figure 2E**, where *S_1/8_* exhibited the largest uncertainties. For Rx, while the mean values for head scans were between 0.4 and 0.5 degree, those for thorax and pelvis scans were all less than 0.4 degree.

**Table 2:** Performance comparison of CNR, Tx and Rx across different protocols.

	Projection No	Dose (mGy)	CNR	Tx (mm)	Rx (degree)
Head					
*Full-dose*	500	3.170	**5.381 ± 1.287 (3.407–7.423)**	\	\
*S_1/2_*	250	1.585	**4.327 ± 0.720 (3.173–5.363)**	0.298 ± 0.208 (0.060 - 0.686)	0.421 ± 0.309 (0.124 - 1.131)
*S_1/4_*	125	0.793	2.706 ± 0.218 (2.420–3.099)	0.315 ± 0.193 (0.058 - 0.643)	0.474 ± 0.396 (0.079 - 1.363)
*S_1/8_*	63	0.399	1.459 ± 0.265 (1.030–1.877)	0.381 ± 0.197 (0.155 - 0.709)	0.477 ± 0.417 (0.057 - 1.266)
Thorax					
*Full-dose*	895	3.970	**3.730 ± 0.704 (2.926–5.260)**	\	\
*S_1/2_*	448	1.987	**3.052 ± 0.501 (2.464–4.083)**	0.365 ± 0.314 (0.088 - 1.544)	0.148 ± 0.076 (0.010 - 0.278)
*S_1/4_*	224	0.994	2.268 ± 0.317 (1.925–2.870)	0.360 ± 0.383 (0.042 - 1.870)	0.176 ± 0.080 (0.077 - 0.466)
*S_1/8_*	112	0.497	1.423 ± 0.262 (1.146–1.974)	0.518 ± 0.339 (0.069 - 1.639)	0.248 ± 0.107 (0.114 - 0.536)
Pelvis					
*Full-dose*	895	3.970	**4.226 ± 1.477 (2.457–6.648)**	\	\
*S_1/2_*	448	1.987	**3.647 ± 1.144 (2.401–5.557)**	0.648 ± 0.292 (0.241 - 1.283)	0.199 ± 0.071 (0.082 - 0.283)
*S_1/4_*	224	0.994	2.846 ± 0.789 (1.905–3.922)	0.751 ± 0.431 (0.167 - 1.984)	0.218 ± 0.119 (0.037 - 0.476)
*S_1/8_*	112	0.497	1.950 ± 0.487 (1.258–2.777)	1.084 ± 0.752 (0.382 - 2.789)	0.341 ± 0.165 (0.086 - 0.657)

1) CNR, Tx, and Rx values were expressed in mean ± SD (minimum - maximum); 2) bold numeric to indicate mean CNR values > 3.0.

## 4 Discussion

Reducing imaging dose of CBCT has been a prominent concern due to the potential health risks associated with recurrent CBCT scans over the entire treatment course. A direct strategy to reducing dose is to obtain projections as few as feasible through expanding the angular gap in sampling, but with the challenge of ensuring that image quality and registration accuracy are not compromised. In the meantime, how to effectively evaluate the impact of projection reduction on image quality and registration accuracy in the clinical context without impairing treatment accuracy is a challenging issue as well.

Our study proposed a virtual clinical trial-based strategy to perform comprehensive clinical evaluation of projection-reduced low-dose CBCT for IGRT application. In this work, we enrolled a total of 71 real-world patients to quantitatively evaluate image quality and registration accuracy for head, thorax and pelvis CBCT scans. Hence, this virtual clinical trial-based study is more accurate in reflecting the clinical context than previous studies that are either highly controlled (20, 21) or phantom-based (15–19). In the meantime, we used the validated open-source research toolkit for image reconstruction (TIGRE-VarianCBCT) and registration (3D Slicer), justifying the reproducibility of all the results. In this context, our analysis yielded important insights.

Firstly, while the reduction of projection numbers led to increased image noise and strike artifacts, acceptable clinical image quality was maintained up to a decrease by half from the default projection number. This was justified by the CNR results in **Table 2**, demonstrating the potential to reduce the imaging dose without significant loss in image quality. However, as the projection number further decreased to 1/4 and 1/8 of the full dose, the CNR values fell below the acceptable threshold, implying substantial deterioration in soft-tissue contrast. This also indicates a limitation in the extent of projection-reduced low-dose CBCT while preserving adequate imaging quality.

Secondly, our study showed that the relative registration errors of projection-reduced CBCT scans compared to full-dose scans were negligible across six directions with mean error< 0.5 mm in translation and<0.4 degree in rotation. Moreover, the error magnitude was< 1.0 mm in translation (Tx) and< 0.4 degree in rotation (Rx). This further demonstrates that a reduction in projection numbers does not necessarily impair the registration accuracy and suggest the maintenance of registration accuracy even at reduced doses, the key concern in IGRT. This finding is in line with those in phantom-based studies by Men et al. (18) and Olch et al. (19). Also, as the projection number was further reduced, slight increase in uncertainties were observed, especially in the LNG direction in both thorax pelvis scans. This phenomenon we assume can be attributed to subtle pelvic anatomy changes along the LNG direction.

Moreover, it is worth noting that no statistically significant shift differences were identified between the dose-reduced protocols and the full-dose reference. The consistency of our findings with the literature (19, 21) reinforces the reliability and potential clinical viability of the projection-reduction strategy.

Our study has several limitations. First, we used auto-matching in CBCT-to-CT registration to minimize any personal bias. However, in clinical practice, therapists or physicists are probably to perform couch shifts with manual corrections. There may be some discrepancy here between the research world and how the shifts would look in a true clinical setting. Second, while the strategy of virtual clinical trial facilitated patient enrollment [for instance, only 6 patients were enrolled in the study by Bryce-Atkinson et al (21)], due to the limitation of the IRB approval, we only enrolled adult patients. It is well known that age is a key factor attributable to estimating patients’ risks (13), as it is pediatric patients that will benefit the most from imaging dose reduction. While we believe some findings in this study are also applicable to pediatric patients, further efforts including more extensive clinical trials are required for solid validation. Thirdly, while we used a virtual clinical trial design to ensure clinical applicability, further prospective clinical trials are needed for a more robust and comprehensive validation, especially in combination with other low-dose techniques. Besides, the retrospective study design, scan sites and the dose-reduction method are also limitations.

## 5 Conclusion

Our study indicates that while the impact of dose-reduction on CBCT couch shifts is not significant, the impact on CNR values is significant. Further validation on optimizing CBCT imaging dose is required.

## Data availability statement

The original contributions presented in the study are included in the article, further inquiries can be directed to the corresponding authors.

## Ethics statement

The studies involving humans were approved by Peking University Cancer Hospital. The studies were conducted in accordance with the local legislation and institutional requirements. Written informed consent for participation was not required from the participants or the participants’ legal guardians/next of kin in accordance with the national legislation and institutional requirements.

## Author contributions

MW: Writing – original draft. KY: Writing – original draft. YZ: Writing – review & editing. JG: Writing – review & editing. XZ: Writing – review & editing. ZL: Writing – review & editing. YL: Writing – review & editing. HW: Writing – review & editing. YD: Writing – review & editing.
